# Case report: A rare case of omental extrarenal rhabdoid tumor and review of the literature

**DOI:** 10.3389/fonc.2024.1341506

**Published:** 2024-05-13

**Authors:** Hui Li, Xiao-Hui Wen, Xiao-Yun Fu, Zuo-Hui Wu

**Affiliations:** ^1^ Department of Ultrasound, Affiliated Hospital of Zunyi Medical University, Zunyi, Guizhou, China; ^2^ Department of Ultrasound, Suining Central Hospital, Suining, Sichuan, China; ^3^ Department of Critical Care Medicine, Affiliated Hospital of Zunyi Medical University, Zunyi, Guizhou, China; ^4^ Department of Critical Care Medicine, Suining Central Hospital, Suining, Sichuan, China

**Keywords:** greater omentum tumor, extrarenal rhabdoid tumor, extrarenal rhabdoid tumor of greater omentum, ultrasound, omentum

## Abstract

Extrarenal rhabdoid tumor of the greater omentum is extremely rare, with only sporadic reports and limited documentation of its ultrasonographic findings. Here, we report a case of an extrarenal rhabdoid tumor of the greater omentum in a 16-year-old girl and review the relevant literature. It was found that the disease mainly occurred in female children and adolescents, and mainly manifested as lower abdominal pain and a large abdominal cystic or solid hemorrhagic mass. The clinical characteristics include a high degree of malignancy and mortality. Ultrasound shows some malignant features, but it is not specific; thus, it is easy to be misdiagnosed in the clinic.

## Introduction

Rhabdoid tumors are rare clinical malignant tumors, which occur widely, mostly in the kidney, but also in organs other than the kidney, collectively referred to as extrarenal rhabdoid tumor (1). The greater omentum is a common site of metastatic tumors, but primary tumors of the greater omentum are very rare (2). Primary extrarenal rhabdoid tumor in the greater omentum is extremely rare, and only sporadic reports have been documented in the literature. Because little is known about it, it is easily misdiagnosed as another type of tumor.

This paper reports a case of extrarenal rhabdoid tumor of the greater omentum treated in our hospital, and discusses its clinicopathological features and differential diagnosis by reviewing the publications, in order to further understand the disease and its diagnosis.

## Case presentation

A 16-year-old girl was hospitalized with severe pain that had developed from recurrent abdominal pain lasting over 10 days. The patient had been experiencing left lower abdominal pain for more than 10 days. The pain was localized and improved after rest without additional obvious symptoms, e.g., chills and fever, nausea and vomiting, diarrhea, hematochezia, and other discomfort. Seven days before the hospitalization, color ultrasound done in the other clinic revealed a pelvic mass of approximately 13 × 10 cm. After taking anti-inflammatory oral pills, the symptoms slightly improved and the pain was tolerable. Half a day before the admission to the hospital, the abdominal pain worsened, accompanied by malignant vomiting and palpitation, and the patient was admitted to the emergency department. Through anal finger examination and abdominal pressing, the huge abdominal mass is palpated to the two transverse fingers on the navel, reaching the midaxillary line on both sides. The boundary of the pelvic mass is clear, and the pain in the left lower abdomen is obvious during compression. The uterus is not palpable.

**Figure 1:**
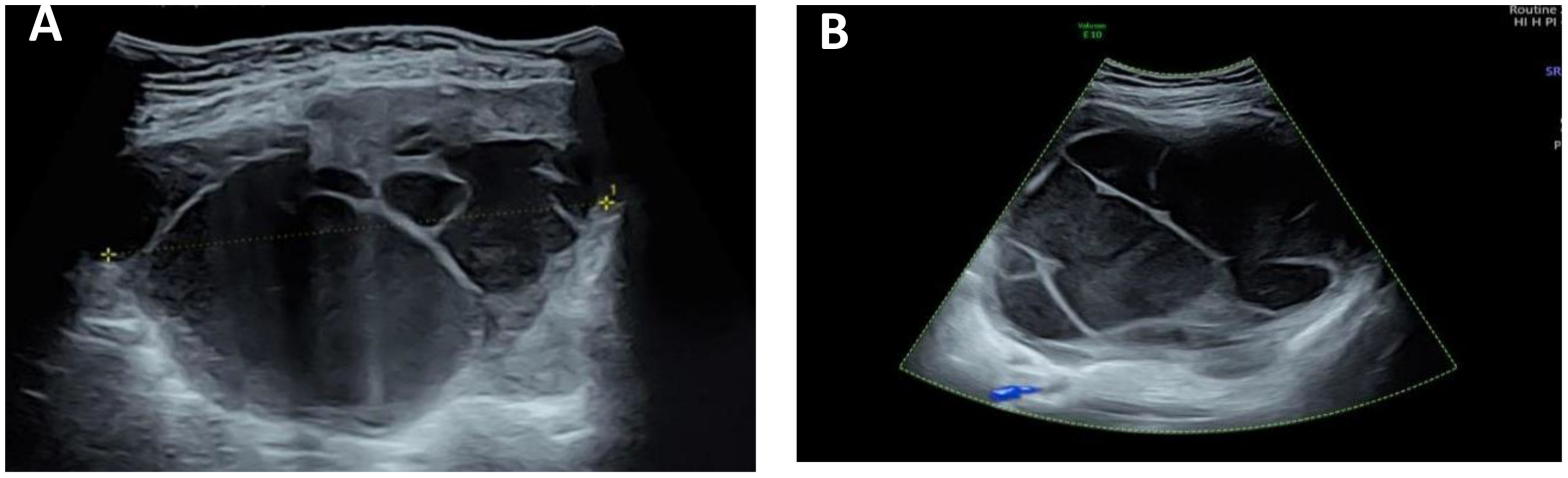
Ultrasound image of the tumor. **(A)** There is a separation in the mass, and the wall thickness is not smooth. **(B)** A weak light spot is seen in the mass, and there is no blood flow signal in the mass.

Color ultrasound on admission suggested that there was no obvious abnormality in uterus and ovary. A cystic solid mass of approximately 13 cm × 11 cm × 15 cm was found in front of the uterus. The wall of the cyst was locally thickened and not smooth with the thicker side measured approximately 0.8 cm. The light bands in different directions could be seen in the cyst, the sound transmission difference of cyst fluid was accompanied by dense and weak light spots, the boundary of the mass was clear, and the shape was not irregular. There were no obvious blood flow signals in the cyst wall and cyst (**Figure 1**).

Laboratory examination: white blood cell 12.1 ×10^9^/L,neutrophil count 9.36 ×10^9^/L, HR hemoglobin 112 g/L, and whole blood hypersensitive C-reactive protein 58.91 mg/L; carbohydrate antigen 12–5 1033 U/mL; postmenopausal Rome index 73.47%; six items of blood coagulation function: plasma fibrinogen 7.12g/L, D-dimer 8.43μg/mL, and fibrinogen degradation products 27.55μg/mL. The liver and kidney functions and infectious markers were normal.

**Figure 2:**
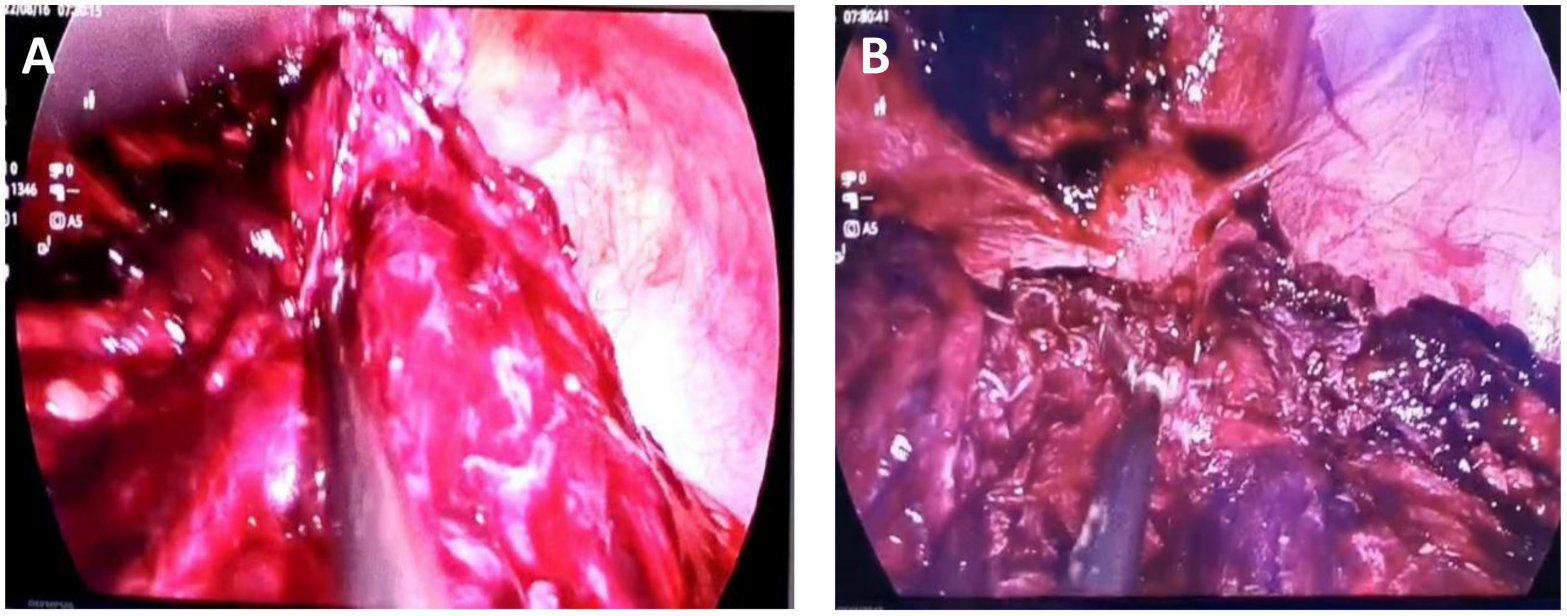
Seen during laparoscopy. **(A)** The omental tumor was large and ruptured bleeding was visible. **(B)** The uterus can be seen behind the omental mass, and metastatic nodules can be seen on the abdominal wall.

The possibility of pedicle torsion of ovarian tumor is considered in the preliminary clinical diagnosis. The patient underwent transumbilical laparoscopic exploration under general anesthesia. Laparoscopic examination during the operation showed that the dense adhesion of the greater omentum and intestine formed an approximately 15 cm × 15 cm fillet-like mass, purplish red, with bleeding and necrosis (**Figure 2**). The mass adheres densely to the left abdominal wall and does not move. There is dark red blood accumulation in the mass, approximately 1,000 mL. After separating the adhesive band between the mass and the left abdominal wall, there was obvious pelvic congestion, and the mass adhered to the left anterior wall of the uterus. There was no obvious abnormality in the appearance of uterus and bilateral fallopian tubes and ovaries. When exploring the greater omentum, there were nodules scattered between 1.0 cm × 0.8 cm and 4 cm × 4.5 cm in size. The nodules were tough and purplish red, and the blood touch was obvious.

Intraoperative freezing suggested that epithelioid cells were found in “pelvic tissue” with necrosis and tended to be malignant to be analyzed with the paraffin section and immunohistochemistry. After consultation with gastrointestinal surgeons, the patient was treated with omentectomy, electroresection of rectal lesions, ovarian biopsy under direct vision, laparoscopic pelvic adhesion lysis, intestinal adhesion lysis, and abdominal puncture and drainage. During the operation, pelvic adhesion, intestinal adhesion, and pelvic congestion were obvious, and pelvic hemorrhage was approximately 1,500 mL.

**Figure 3:**
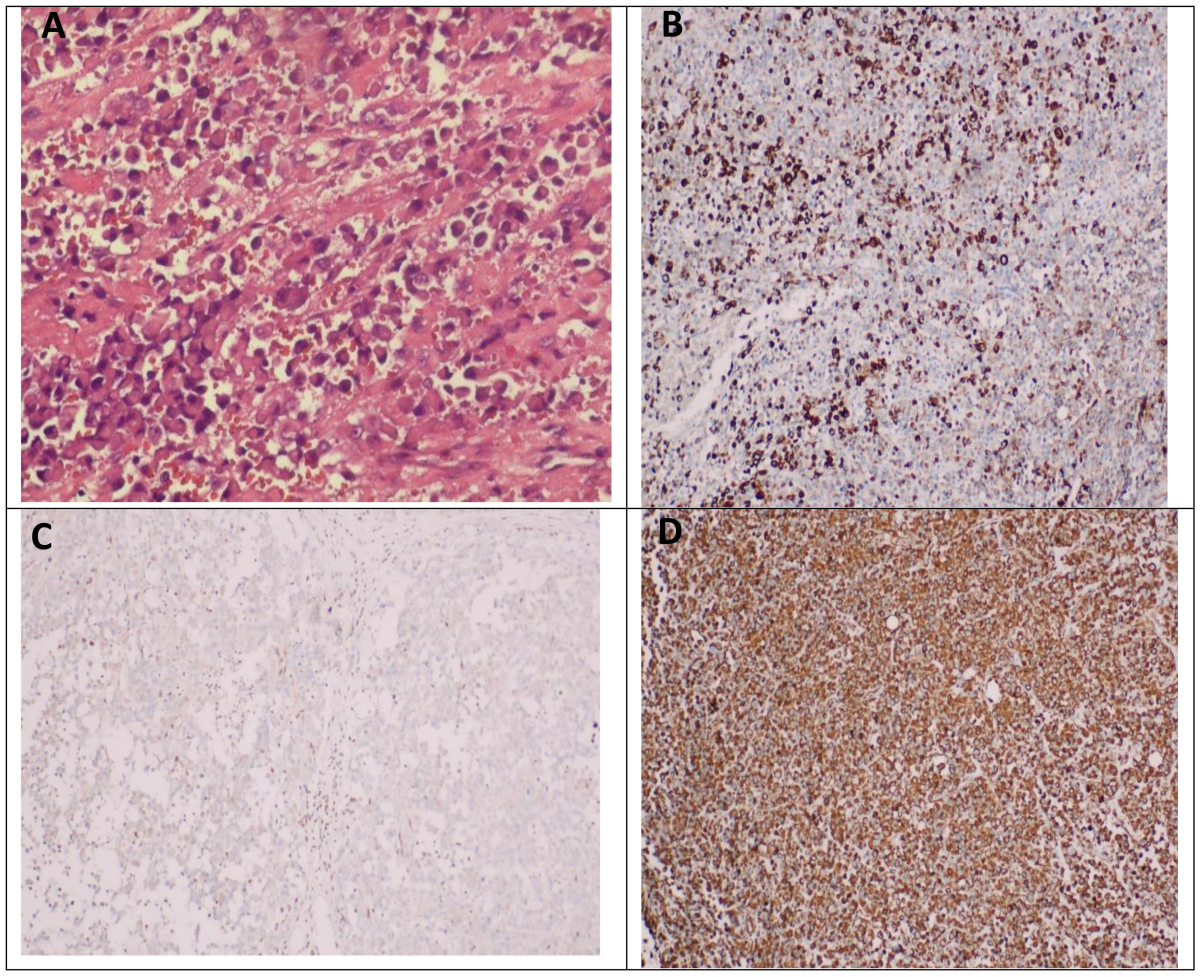
Pathological manifestations of extrarenal rhabdoid tumor of the greater omentum. **(A)** Microscopically, the tumor showed solid flake and nest distribution, infiltrative growth, round or oval tumor cells, rich cytoplasm, eosinophilia, large nucleus, deviation, vesicular chromatin, obvious nucleoli, easy mitosis, and poor adhesion of tumor cells in some areas. It can be seen that the rhabdomyosus-like cells with nuclear deviation and strong eosinophilic cytoplasm have necrosis. **(B)** Immunohistochemical results showed CK-pan positive in tumor cells. **(C)** The immunohistochemical results showed that the tumor cells were negative for INI1. **(D)** The results of immunohistochemistry showed that the tumor cells were Vimentin positive.

Postoperative pathological examination showed that malignant tumors were found in “pelvic tissue”, “greater omentum”, “mesentery of small intestine”, “abdominal wall mass”, and “anterior rectal wall mass”. The “bilateral ovarian biopsy tissue” showed ovarian cortex under the microscope, and no tumor was found. Microscopically, the tumor showed solid flake and nest distribution, infiltrative growth, round or oval tumor cells, rich cytoplasm, eosinophilia, large nucleus, deviation, vesicular chromatin, obvious nucleoli, easy mitosis, and poor adhesion of tumor cells in some areas. It can be seen that the rhabdomyoid cells with nuclear deviation and strong eosinophilic cytoplasm have necrosis. The results of immunohistochemistry showed the following: Bcl-2(-), CD34(-), CD45(LCA)(-), CK(AE1/AE3)(+), CK20(-), CK7(-), CR(calretinin)(-), Desmin(-), ERG(-), HMB45(-), INI1(-), S-100(-), SMA(-), TLE1(-), Vimentin(+), and WT1 (Wilm’s tumor)(-). After the general discussion in the Department of Pathology, combined with the results of immunohistochemistry, the malignant tumor of the greater omentum was consistent with the malignant extrarenal rhabdoid tumor (**Figure 3**).

After operation, it was diagnosed as rupture of a malignant extrarenal rhabdoid tumor with hemorrhage and necrosis. No other primary cancers or metastases were found by PET-CT after operation. The patient was hospitalized again half a month after discharge. She was given symptomatic support treatment because of cancerous persistent blood loss and infection. She died 1 month after operation.

## Discussion

Renal rhabdoid tumor is a new subtype that was isolated from nephroblastoma and first reported by Beckwith in 1978 (3). Later, it was reported in other organs other than the kidney, including the heart (4), lung (5), liver (6), orbit (7), intestinal tract (8), vulva (9), and central nervous system (10). According to the location of the tumor, it can be divided into three types: malignant rhabdoid tumor of the kidney (MRTK), central nervous system atypical teratoid/rhabdoid tumor, and extrarenal extracranial rhabdoid tumor (EERT) (11). Malignant rhabdoid tumors are common among infants and children, and the non-kidney ones have a wide range of age and can occur in adults, but there are more cases in infants and children; EERT cases are mostly common among late adolescence or young people (12).

The pathogenesis of extrarenal rhabdoid tumor is unclear. Eighty percent of the cases showed 22q11.2–12.2 deletion. The deletion or mutation of tumor suppressor gene SMARCB1/hSNF5/INI1 may play a role in the pathogenesis of extrarenal rhabdoid tumor (13). At present, the immunohistochemical results of almost all rhabdoid tumors show that INI-1 is negative, which has been used as one of the indicators for the diagnosis of rhabdoid tumors. Rhabdomyoid cells can be found in many kinds of tumors. The expression of INI-1 gene or INI-1 protein can be detected by immunohistochemistry as a marker for differential diagnosis of rhabdoid tumors and other tumors. With further research, researchers have proposed multiple hypotheses about the tissue origin of the tumor cells, such as primitive mesenchymal cells, neuroectodermal cells, and epithelial cells (14), without strong evidence. Hence, they are still classified as tumors of unknown tissue origin.

Most of the clinical features of extrarenal rhabdoid tumor are local mass, combined with compression or invasion of adjacent tissue. The general symptoms of greater omentum tumor are abdominal discomfort (45.5%), abdominal mass (34.9%), and abdominal distension (15.2%). Early adjacent tissue invasion or distant metastasis may occur. The prognosis is very poor, the mortality rate is high, and the 5-year survival rate is <50% (15). This patient presented with a pelvic mass with intermittent abdominal pain for 10+ days. The cause of abdominal pain may be related to the hemorrhage and necrosis of the mass. Adjacent tissue invasion and intestinal metastasis occurred at the time of onset, and the course of the disease progressed rapidly. She died in only 1 month.

The radiological features of extrarenal rhabdoid tumors were as follows: most of the tumors were large, showing solid or cystic-solid mixed masses; the distinction between cystic and solid was unclear; MRI mainly showed that inhomogeneous T1WI showed iso-signal or slightly low signal; T2WI showed high signal or slightly high signal; and solid part DWI showed high signal, inhomogeneous enhancement on the enhanced scan, and no enhancement in the cystic or necrotic area. Some of the lesions were associated with calcification, bleeding, local invasion, or distant metastasis (16, 17).

Extrarenal striated muscle of the greater omentum is very rare. There are only sporadic reports. We systematically reviewed the literature on extrarenal rhabdomyoma of the greater omentum by searching databases at home and abroad, i.e., PubMed, Zhiwang, and Wanfang, using keywords including omental tumor, extrarenal rhabdomyoma, and omental extrarenal rhabdoid tumor. As of August 2023, a total of three cases have been retrieved, and our case is the fourth one (**Table 1**).

**Table 1:** Clinical and imaging features of omental extrarenal rhabdoid tumor reported in the literature.

Case no.	1	2	3	4
Author	Wang Juan	So-Hyun Nam, et al.	Leonardo Suryawan, et al.	Li Hui, et al.
Year of publication	2008	2014	2023	This study
Age	15 years old	10 years old	9 years old	16 years old
Gender	Female	Female	Female	Female
Symptom	Lower abdominal tenderness, rebound pain, shifting dullness (+)	Lower abdominal pain	Abdominal swelling, pain and anemia, nausea, constipation, shifting dullness (+)	Lower abdominal pain, nausea and vomiting, palpitation
Ultrasound	Solid space on the left side of the cervix, abdominal and pelvic effusion	Large multi-segmental hematoma, enhanced peripheral blood vessels, and a small amount of hemorrhage in the abdominal cavity	N/A	There is a multilocular cystic space in front of the uterus with a weak spot
CT	N/A	Lobulated hemorrhagic mass, irregular enhancement around, and a small amount of hemorrhage in the abdominal cavity	Inhomogeneous enhancement of omental mass. Peritoneal effusion	N/A
Size	8 × 8 × 6 cm	9 × 6 × 6.5 cm	8 × 6 × 8 cm	13 × 11 × 15 cm
Intraoperative bleeding	1,500 mL	N/A	3,000 mL	1,500 mL
Result	Died 2 months later	Died 9 months later	Died 3 weeks later	Died 1 month later
Distant metastasis	Yes	N/A	N/A	Yes

N/A, Not applicable; (+), It indicates that the shifting dullness is positive.

**Table 1** shows the clinical and imaging features of omental extrarenal rhabdoid tumor reported in the literature. The disease seems to be an adolescent disease. The maximum age of onset is 16 years old, the youngest age is 9 years old, the average age is 12.5 years old, the sex preference seems to be female, and all four patients are female. The clinical manifestations were lower abdominal pain; one had nausea, constipation, and anemia; one had rebound pain; and one had nausea, vomiting, and palpitation. Three cases had abdominal effusion before operation. All the masses had hemorrhage and necrosis. Except for one case without intraoperative bleeding, the intraoperative bleeding volume was larger in the other three cases, with the largest being 3,000 mL and the smallest being 1,500 mL. According to the longest diameter of the mass, the average size of the mass was approximately 10 cm, the largest mass was approximately 15 cm, and the smallest was approximately 8 cm. Most of the masses were hemorrhagic masses, including solid in one case, lobulated hemorrhagic mass in one case, cystic hemorrhagic mass in one case, and a mass of unknown nature in one case. Inhomogeneous mass enhancement was found in two cases by CT (18, 19), and CT scan was not performed in the other two cases. All patients received surgical resection. During the operation, one patient found that there were 24 lymph nodes with metastasis in 25 lymph nodes, one patient found greater omentum with pelvic and abdominal implants, and the other two cases had no lymph node or distant organ metastasis. Because of their poor condition, three patients did not receive radiotherapy and chemotherapy and died 3 weeks, 1 month, and 2 months after operation. Only one 10-year-old child received chemotherapy with a VDC/IE (VCR, doxorubicin, and cyclophosphamide/ifosfamide and etoposide) regimen. Although the VDC/IE regimen underwent four cycles of chemotherapy, she died 9 months after the operation because of the deterioration of the disease.

Ultrasonographic findings of extrarenal rhabdoid tumor are rarely reported. Of the two previously reported cases of extrarenal rhabdomyoma of the omentum and kidney, one case showed solid space occupying on the left side of the cervix (20), one case showed large abdominal hematoma (19), and both cases had peritoneal effusion. In our case, ultrasound revealed a huge tumor in the pelvic cavity with clear boundary and regular shape, mainly cystic, uneven thickening of the cyst wall, less smooth, multiple septum and dense weak light spots in the cyst, and no blood flow signal in the intracapsular and cyst wall. The dense and weak light spots in the capsule indicated that there is bleeding in the tumor. According to the consensus guide for the O-RADS ultrasound risk stratification and management system (21) published by the ovarian-adnexal reporting and data system (O-RADS), the mass in this case is larger than 10 cm in diameter, with an unsmooth inner wall, irregular septum, multilocular cyst, and a blood flow score of less than 4 points, which is in accordance with the O-RADS4 category, with a moderate malignant risk of 10% to 50%.

The ultrasonographic features of this case of omental extrarenal rhabdoid tumor should be differentiated from pelvic inflammatory mass, chocolate cyst, cystadenoma, and so on. (1) Pelvic inflammatory mass: mostly acute or recurrent history of pelvic infection. It is more common among sexually active people. In addition, it usually manifests as a lower abdominal pain without periodicity and possibly accompanied by a fever and leukocytosis, which can be subdued by antibiotic treatment. When hydrosalpinx or empyema occurs, it can be shown as a cystic mass like a pelvic intestine or a circuitous tube, with a uniform thickness of the cyst wall and thin light spots in the cyst. When pelvic empyema occurs, a cloud-like low echo area with an irregular shape and an uneven echo can appear in the pelvic cavity. The patient is a teenager without sex. Moreover, she did not have symptoms of fever and chills. (2) Chocolate cyst:Most of the patients had a history of dysmenorrhea, with round or oval cysts in the pelvis, which could be single or multiple; the outer edge of the cyst wall was clear, the inner wall was rough, and cloud-like dense light spots or irregular hyperechoic masses could be seen in the cyst. (3) Ovarian serous cystadenoma: most of the cysts were single-chamber cysts with a thin wall and a smooth inner wall, common nipple growth in multi-chamber cysts, and serous fluid in the cysts and transparent sound. (4) Ovarian mucinous cystadenoma: most of them are large multilocular cysts containing jelly-like mucus and clear fluid. Most of the cystadenoma show cloud-like light spots and nipples are rarely seen in the capsule.

## Conclusion

Extrarenal rhabdoid tumor of the greater omentum have the characteristics of high degree of malignancy, poor prognosis, and high mortality. Ultrasound has certain malignant features, but it cannot determine with certainty the source and characteristics of the lesion. Therefore, contrast-enhanced ultrasound can further determine the blood flow of the tumor and the relationship between the tumor and its periphery. For the diagnosis, we should consider both the typical histological characteristics of rhabdomyoid cells and the age of the patient. Because of the negative INI-1 protein expression in immunohistochemistry, when we detect that the abdominal cystic solid mass in children or adolescents has the characteristics of malignant tumor but bilateral ovaries are normal, we should suspect the disease, so as to provide more imaging evidence for clinical diagnosis and treatment.

## Data availability statement

The original contributions presented in the study are included in the article/supplementary material. Further inquiries can be directed to the corresponding author.

## Ethics statement

The studies involving humans were approved by Ethics Committee of Suining Central Hospital. The studies were conducted in accordance with the local legislation and institutional requirements. Written informed consent for participation in this study was provided by the participants’ legal guardians/next of kin. Written informed consent was obtained from the minor(s)’ legal guardian/next of kin for the publication of any potentially identifiable images or data included in this article.

## Author contributions

H-L: Writing – review & editing, Writing – original draft, Data curation. X-HW: Writing – review & editing, Resources, Data curation. X-YF: Data curation, Resources, Writing – review & editing, Supervision, Project administration. Z-HW: Writing – review & editing, Supervision, Project administration.
